# LC–MS/MS assay for quantitation of enalapril and enalaprilat in plasma for bioequivalence study in Indian subjects

**DOI:** 10.4155/fsoa-2016-0071

**Published:** 2017-02-02

**Authors:** Dhiman Halder, Shubhasis Dan, Murari Mohun Pal, Easha Biswas, Nilendra Chatterjee, Pradipta Sarkar, Umesh Chandra Halder, Tapan Kumar Pal

**Affiliations:** 1 Bioequivalence Study Centre, Department of Pharmaceutical Technology, Jadavpur University, Kolkata, India; 2 Organic Chemistry Section, Department of Chemistry, Jadavpur University, Kolkata, India; 3 TAAB Biostudy Services, Kolkata 700032, India

**Keywords:** bioequivalence study enalapril, enalaprilat, human plasma, LC–MS/MS, protein precipitation

## Abstract

**Background:**

Enalapril (EPL) is an angiotensin-converting enzyme inhibitor for the treatment of hypertension and chronic heart failure. Enalaprilat (EPLT) is an active metabolite that contributes to the overall activity of EPL.

**Aim:**

To quantitate EPL along with its metabolite EPLT using LC–MS/MS, a bioanalytical method was developed and validated with tolbutamide in human plasma using a protein precipitation technique.

**Results:**

The sensitive and selective method has an LLOQ of 1 ng/ml with a linearity range of 1–500 ng/ml for both EPL and EPLT using 300 µl of plasma without any matrix effect.

**Conclusion:**

Linearity, specificity, accuracy, precision and stability, as well as its application to the analysis of plasma samples after oral administration of 20 mg of EPL maleate in healthy volunteers demonstrate applicability to bioavailability/bioequivalence studies.

## Lay abstract

The present study describes a sensitive, selective, simple, accurate and reproducible LC–MS/MS method for the simultaneous determination of both enalapril (EPL) and enalaprilat (EPLT) in human plasma. The results obtained indicate the high sensitivity of the described method for analysis of EPL and EPLT, which render this method particularly useful for pharmacokinetic or bioequivalence studies. The proposed method has been applied for the analysis of EPL and EPLT in the plasma of healthy volunteers in a single-dose pharmacokinetic study.

**Figure 1.**
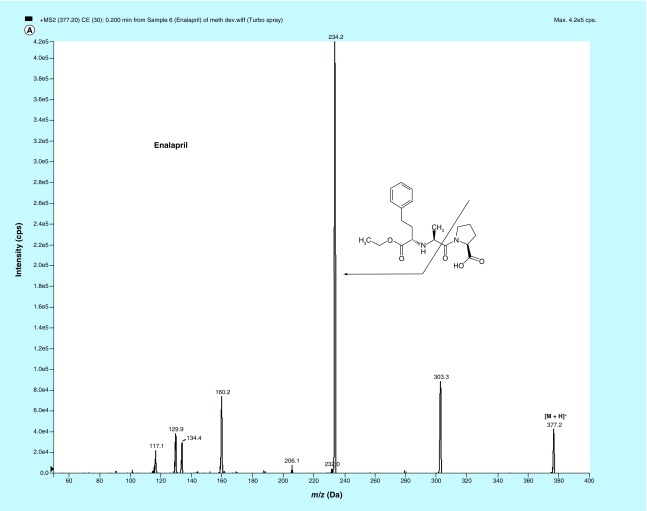

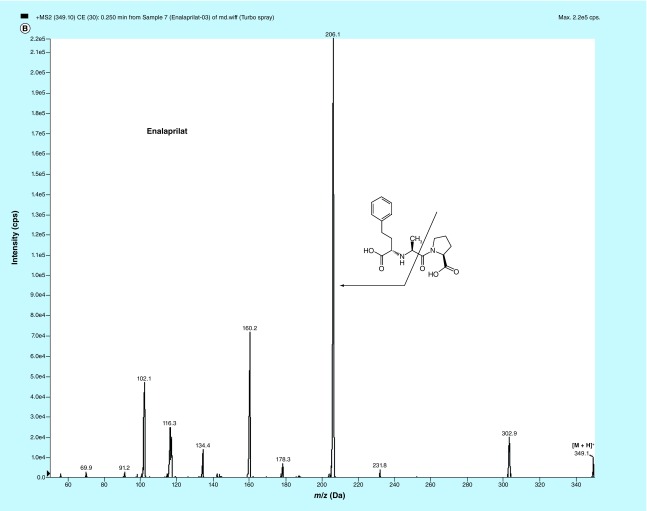

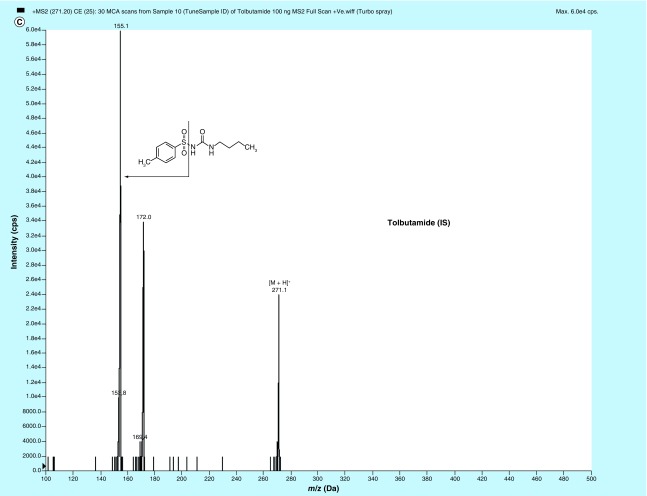
**MS/MS fragmentation pattern of (A) enalapril (EPL, (B) enalaprilat (EPLT) and (C) tolbutamide (TBM).**

**Figure 2.**
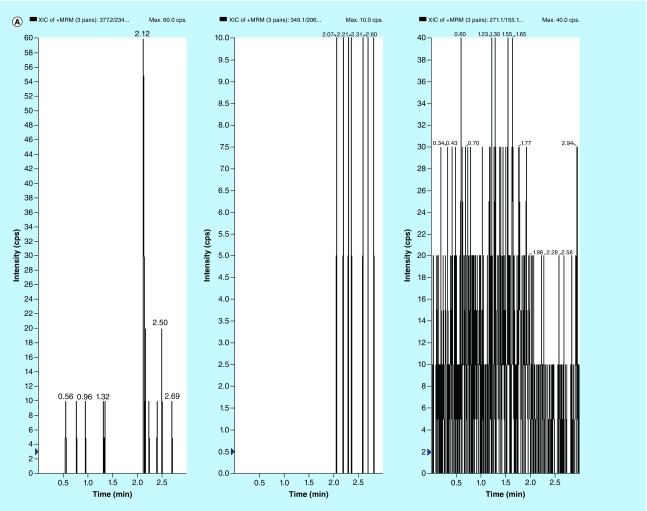

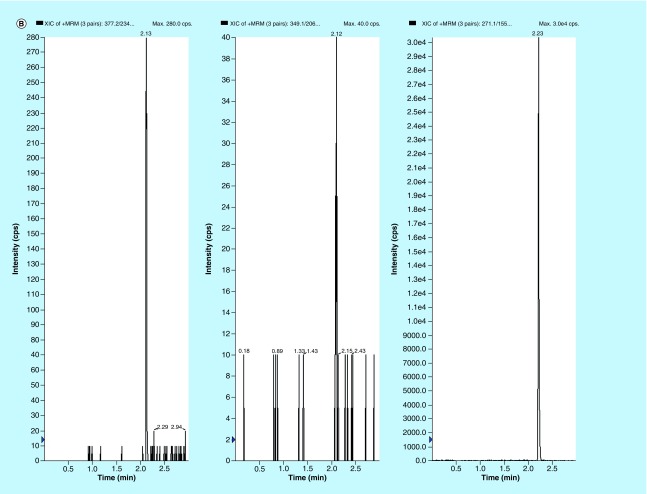

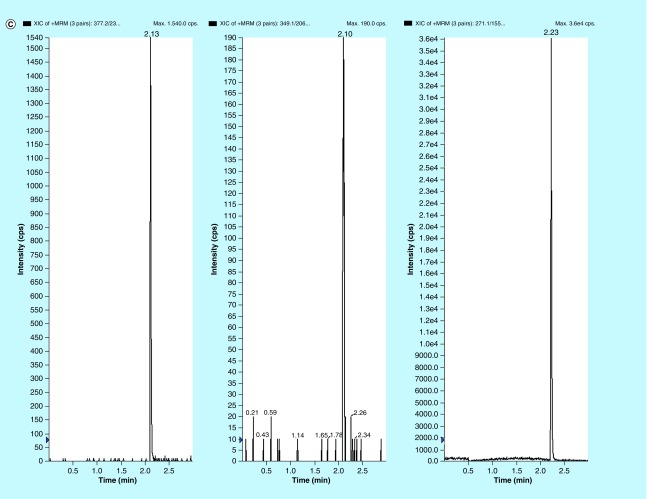
**Typical MRM chromatograms of EPL, EPLT and TBM (IS) in (A) human double blank plasma, (B) human blank plasma with IS and (C) human blank plasma spiked with EPL and EPLT at LLOQ (1 ng/ml) with IS.**

**Figure 3.**
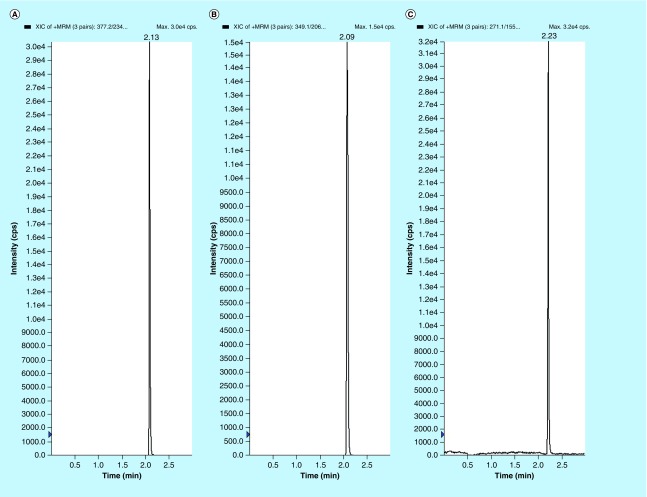
**Representative MRM chromatograms of volunteer plasma samples of (A) EPL, (B) EPLT and (C) TBM (IS) spiked in human volunteer plasma at 100 ng/ml.**

**Figure 4.**
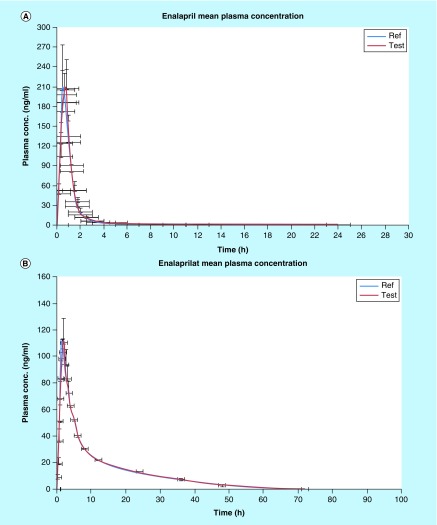
**Graphical representation of mean plasma concentration versus time of (A) EPL and (B) EPLT following 20 mg single oral dose of test and reference preparation to 30 healthy human volunteers.**

Hypertension and cardiovascular disease are a major cause of morbidity in India and abroad. Antihypertensives are a class of drugs that are used to treat hypertension (high blood pressure) [1,2] and cardiovascular disease [3,4]. Enalapril maleate [(S)-1-(N-(1-(ethoxycarbonyl)-3-phenylpropyl)-l-alanyl)-l-proline, (Z)-2-butenedioate salt] is a salt of both enalapril (EPL) and maleic acid [1,5–7]. EPL is a potent antihypertensive drug, a vasodialator and also an angiotensin-converting enzyme (ACE) inhibitor [5,8–13] which is widely used for the treatment of hypertension and congestive heart failure [1,4,14–17]. In the kidneys, rennin is synthesized and after release into the circulation it acts on a plasma precursor for the production angiotensin I. ACE is a peptidyl dipeptidase which catalyzes the conversion of angiotensin I to angiotensin II, a potent vasoconstrictor substance that stimulates the release of aldosterone from the adrenal cortex and also increases endogenous bradykinin concentrations [16]. Thus, there is an increase in blood pressure and cardiac failure. ACE inhibitors such as EPL inhibit the conversion of angiotensin I to angiotensin II decreasing blood pressure and aldosterone secretion, slightly increasing serum potassium levels, and causing sodium and fluid loss. Also, increased prostaglandin synthesis is involved in the antihypertensive action [6–7,13,18–19].

After absorbtion from the gastrointestinal tract, EPL is hydrolyzed of its ethyl ester, in other words, it is de-esterified *in vivo* to its active metabolite enalaprilat (EPLT) by hepatic metabolism in the liver. EPLT is the only metabolite of EPL [1,16,18,20–21]. It is a more potent and effective ACE inhibitor than EPL and is also effectively used in the treatment of hypertension and congestive heart failure through dilation of peripheral vascular resistance without causing any significant changes in heart rate or cardiac output [14,18,22]. After oral administration of EPL, maximum plasma concentration is reached within about 1 h [14,18,22–23]. In healthy subjects EPL is rapidly absorbed from the GI tract and bioavailability is about 60–70%. The terminal half-life of EPL is approximately 2 h [11,14,18,22–23]. The absorption of EPL after oral administration is not influenced by the presence of food in the GI tract [11,18]. However, after 4 h it is not detected above 10 ng/ml [14,22]. EPLT is detectable for upto 72 h and maximum plasma concentration is observed at approximately 3–4 h after administration with a half-life of approximately 30–35 h [14,18,22–23]. When administered orally, EPLT is poorly absorbed. Primarily, the excretion of EPL is renal. In the urine and feces, approximately 94% of the dose is recovered as EPL or EPLT. Also, in urine EPLT is the major components which accounting for about 40% of the dose [18].

From literature review we have found several bioanalytical methods of both HPLC and MS for the simultaneous quantification of EPL and EPLT in human plasma [7,14,16–18,20,22–25]. These methods describe the pharmacokinetics or bioequivalence studies of both EPL and EPLT after administration of the drug enalapril maleate in the human body. An LC–MS method by Yoon *et al.* reported that after oral intake the mean value of C_max_ is 102 ± 39 ng/ml and T_max_ is 0.96 ± 0.36 h [7]. A GC–MS method demonstrated that after single oral administration of 20 mg of EPL to 24 healthy adult subjects pharmacokinetic parameters (mean ± SD) are 72.9 ± 41.6 ng/ml (C_max_), 4.0 ± 1.4 h (T_max_), 4.2 ± 1.6 h (T_1/2_) etc. After a single oral administration of 20 mg EPL maleate to 12 healthy volunteers C_max_ are 152.1 ng/ml (EPL) and 88.5 ng/ml (EPLT), respectively [20]. Another LC–MS/MS method reported that the maximum plasma concentration (C_max_) are 90.5 ± 28.4 and 47.5 ± 12.4 ng/ml, T_max_ are 0.860 ± 0.310 and 4.20 ± 1.06 h, AUC_0–t_ are 136 ± 36 and 401 ± 89 ng/ml h, AUC_0–∞_ are 138 ± 36 and 420 ± 91 ng/ml h and T_1/2_ 1.35 ± 0.61 and 6.71 ± 2.22 h for EPL and EPLT, respectively [24].

So, therefore a new LC–MS/MS method is required for development, validation and simultaneous quantification of EPL along with its active metabolite EPLT in human plasma through a pharmacokinetics or a bioequivalence study. This is owing to the fact that the active metabolite EPLT has low exposure to the central compartment in therapeutic human dose and is detectable in pictogram (LODs are 0.25 and 0.80 ng/ml for EPL and EPLT, respectively), and quantified in nanogram, levels in human plasma. Due to exposure to plasma in therapeutic dose in very low strength (20 mg), it is very difficult to use HPLC only. Hence, to perform pharmacokinetics or bioequivalence studies of this active drug metabolite, in other words, EPLT along with its pro-drug EPL in human plasma, a highly sensitive new LC–MS/MS method is needed. In the present investigation we have developed and validated such a bioanalytical method, which is very simple, fast, rapid and sensitive (LLOQ 1 ng/ml) in human plasma. In addition, this method has been implemented in 30 healthy male, adult, human volunteers under fasting conditions to establish the bioequivalence study for both EPL and EPLT.

## Experimental

### Chemicals, reagents & solvents

The active pharmaceutical ingredient of EPL (99.45%), EPLT (99.95%) and tolbutamide (TBM) (IS, internal standard) were purchased from Clearsynth Labs Pvt. Ltd., (Mumbai, India). HPLC grade methanol and acetonitrile were obtained from Merck Specialties Pvt. Ltd., (Mumbai, India) and guaranteed analytical reagent grade formic acid, chloroform, isopropyl alcohol were also obtained from Merck Specialties Pvt. Ltd, (Mumbai, India). Water used in the entire analysis was prepared from Milli-Q water purification system procured from Millipore (Elix, Milli-Q A10 Academic, MA, USA) until a resistively of 18.2 MΩ was achieved. The blank human plasma with EDTA-K3 anticoagulant was collected from clinical pharmacological unit of Bioequivalence Study Centre, Jadavpur University (Kolkata, India) and was stored at -20°C until use.

### Instrumentation & chromatographic conditions

The LC system (Shimadzu, prominence) equipped with binary pump (LC-20AD), an autosampler set at 15°C (SIL-20A), an online solvent degasser (DGU-20A3) and column oven (CTO-10AS) were used to inject 10 µl aliquots of the processed samples on a C18 analytical column (50 mm × 3 mm, 5 µm, Phenomenex Kinetex). Separation and elution were achieved by gradient system using 0.1% formic acid in methanol and 0.1% formic acid in Milli Q water as the mobile phase, at a flow rate of 0.5 ml/min. The total LC run time was 3 min. Mass spectrometric detection was performed using tandem mass spectrometer API 2000 model (MDS Sciex, Toronto, Canada). LC–MS/MS detector analysis was performed with TurboIonSpray source interface in positive ionization mode. Direct infusion of 100 ng/ml of each analyte in methanol was made by syringe pump to optimize parameters to detect the most vivid signals of transitions from precursor to product ions. Quantification was performed using multiple reaction monitoring mode, based on protonated precursor → product ion transitions for EPL (*m/z* 377.2 → 234.2), EPLT (*m/z* 349.1 → 206.1) and TBM (IS) (*m/z* 271.1 → 155.0). Q1 and Q3 were maintained at unit resolution and dwell time was set at 200 ms. Source-dependent and compound-specific parameters were optimized by monitoring the *m/z* of precursor/product ions (Tables 1 & 2). Analyst software version 1.5 was used for instrument control, data acquisition and data processing. The optimized mass parameters are elaborated in Tables 1 & 2.

### Stock solutions & working standards

Stock solutions of EPL and EPLT and TBM (IS) were prepared by dissolving accurately weighed samples in the methanol to obtain concentrations of 1 mg/ml. The stock solutions were then gradually diluted with diluent, in other words, methanol: water: 50:50 (v/v) to obtain standard working solutions for calibration at 10, 50, 100, 250, 500, 1000, 2500 and 5000 ng/ml for both EPL and EPLT. For preparation of calibration standards and quality control (QC) samples, appropriate aliquots of the stock solution or working solutions were added to drug-free pooled human plasma. The final concentrations were 1, 5, 10, 25, 50, 100, 250 and 500 ng/ml for the calibration standards and 1 ng/ml (LLOQ), 3 ng/ml (low), 200 ng/ml (medium) and 400 ng/ml (high) for QC samples. The working IS solution (TBM 500 ng/ml) was prepared in diluting solvent stated above. All stock solutions (1.0 mg/ml) and QC solutions were stored at -20°C and working standard solutions were stored at 4°C.

### Sample preparation & extraction

Sample preparation was performed by simple protein precipitation method. Working stocks of EPL and EPLT were prepared in 50: 50 (v/v) mixture of methanol-water and spiked in blank plasma to get calibration standard samples. After thorough vortexing 300 µl plasma from each calibration standard sample and QC samples were crashed with 900 µl ice cold MeCN (acetonitrile) containing 1 µg/ml TBM (IS) followed by vortex mixing for 10 min and centrifugation at 10,000 rpm for 15 min. Supernatant was separated and loaded into autosampler vials for LC–MS/MS run. Volunteer samples were crashed and processed following the same procedure.

### Method validation

The method validation was conducted in accordance with the guidelines US FDA or EMA for selectivity, sensitivity, linearity, precision, accuracy, recovery and stability [26–32].

### Selectivity & LLOQ

Selectivity is the ability of an analytical method to differentiate and quantify the analyte in the presence of other components in the sample. The specificity was defined as noninterference of EPL and EPLT at retention times from the endogenous plasma components and lack of cross-interference between analytes and IS using the suggested extraction procedure and LC–MS/MS conditions. The specificity of the method was tested by analyzing six different batches of blank human plasma containing anticoagulant comparing chromatograms of blank plasma with the corresponding spiked plasma samples. LOD was defined as three-times of the S/N and the LLOQ was the lowest standard on the calibration curve at which the response of analytes should be at least five-times the response compared with blank response and back-calculated analytes concentration should have precision not ≤20% of the CV and accuracy within 20% of the nominal concentration. As the method is intended to quantify more than one analyte, each analyte was tested for interference and selectivity was ensured at the LLOQ of the analytes.

### Accuracy & precision

The accuracy of an analytical method represents the closeness of average test results incurred by the method to the actual concentration of the analytes. The precision of the analytical method describes the closeness of repeated individual measures of analytes. The deviation of the mean from the nominal value serves as the measure of accuracy. Mean value should be within 15% of the nominal value except at LLOQ, where it should not deviate by more than 20%. The precision determined at each concentration level should not exceed 15% of the CV except for the LLOQ, where it should not exceed 20% of the CV [26–27,29–32]. The ‘within-run’ precision and accuracy (assessment of precision and accuracy during a single analytical run) was estimated by analyzing five replicates of EPL and EPLT at three different QC levels-low, middle and high (LQC, MQC and HQC) in human plasma.

The ‘between-run’ precision and accuracy (assessment of precision and accuracy over time) was determined by analyzing the three level QC samples on three consecutive days. A total of six replicates of each QC concentration were assayed on day 1, day 2 and day 3. Precision is expressed as the percent CV (% CV), while accuracy is measured as the percent nominal (% nominal).

### Recovery & matrix effect

The recovery of analytes in a particular assay represents the ratio of detector response obtained from the extracted analytes from the biological matrix to the detector response obtained from unextracted analytes. The percentage recoveries were determined by measuring the peak areas of the drug from the prepared plasma at six replicate of LQC, MQC and HQC samples. The peak areas of these QC Samples were compared with the absolute peak area of the unextracted standards containing the same concentrations of the EPL and EPLT.

The matrix effect was determined according to the procedures demonstrated by Matuszewski *et al.* [33] and Ghosh *et al.* [34]. Analytes were spiked in post extraction matrix from six different plasma donors at three QC concentrations. Then matrix effect was calculated as the ratio of peak response obtained from post extraction matrix samples to samples with those of the pure QC standards [35]. Matrix effect of the internal standard was also calculated from the six different lots of plasma at a concentration of 50 ng/ml. Specificity of the method was ensured by using six different sources of plasma sample and comparing chromatograms of blank plasma with the corresponding spiked plasma samples.

### Calibration curves & calculation procedures

On spiking the drug-free plasma samples with eight nonzero calibration working solutions covering the total range of quantitation (1–500 ng/ml conc. in plasma for both EPL and EPLT) along with a blank sample (spiked without drug and IS) and a zero sample (spiked with IS), the calibration curves were generated by plotting the ratios of AUC of analyte versus AUC of IS in y-axis against the calibration concentration ratios of drug to IS in plasma in x-axis by least-squares linear regression method following the equation, y = mx + c, where y represents the peak area ratio of analyte to IS, and x represents the plasma concentration of analyte respectively. The linearity of the method was evaluated using six different calibration curves. The LLOQ was defined as the lowest concentration yielding a S/N of at least 5 with a CV < 20% and accuracy of 80–120%. The acceptance criterion for other standard concentrations was within 15% deviation from the nominal values [26–27,29–32].

### Stability

Stability studies of EPL and EPLT in human plasma were performed with three different concentrations of QC sample (3, 200 and 400 ng/ml) of the analytes each in six replicates. The bench-top stability was performed for each analyte in plasma was determined by three QC levels in six replicates for 8 h at ambient temperature (24 ± 2°C). The autosampler stability of EPL and EPLT were determined periodically by injecting replicate and processed plasma samples after the sample loading up to 24 h at 4°C. For determination of freeze–thaw stability of the analytes, the spiked plasma samples were frozen for 24 h at -20°C and thawed unassisted at room temperature. When completely thawed, the samples were refrozen for 12–24 h at -20°C. On repeating the freeze−thaw cycle for 2 days, bioanalysis was done on the third day. For short-term stability, frozen plasma samples (-20°C) were kept at room temperature for 4 h before sample processing. Long-term stability was evaluated after storing the frozen plasma samples at -20°C for the whole duration of the study which was almost 30 days. The stability of the prepared plasma samples was tested after keeping the processed samples in the autosampler at 4°C for 24 h.

### Application to a bioequivalence study

The developed and validated LC−ESI−MS/MS assay method was applied to compare the bioavailability of two formulations of EPL by conducting the single oral dose, open label, randomized, two period, two sequence, crossover study [27] of 30 healthy Indian volunteers (male) with a mean age of 27.53 years (range 21–34 years), mean body weight of 60.23 kg (range 54–66 kg) and mean body height of 168.30 cm (range 162–174 cm) under fasting condition. After an overnight fasting period (10 h), each volunteer was administered either single dose of test preparation of EPL 20 mg Tablet (containing EPL maleate 20 mg, manufactured by JV LLC ‘Pharmland,’ The Republic of Belarus) or reference preparation Renitec 20 mg Tablet (containing EPL maleate 20 mg, manufactured by Merck Sharp & Dohme B.V., The Netherlands) with 240 ml of water, in the sequence determined by randomization. A wash out period of 21 days had been followed between two consecutive dosing sessions. The protocol (Protocol No. 03/15/296) of the study was accepted by the Drugs Controller General of India, New Delhi, India and approved by the registered Institutional Independent Ethics Committee (Reg. No. ECR/103/Indt/WB/2013), Kolkata, India. Before participation in the study, a written informed consent form was given to all participants. Total 23 blood samples were collected from each volunteer from anticubital vein at 0 h (before drug administration), 0.17, 0.33, 0.5, 0.75, 1.0, 1.42, 1.5, 2.0, 2.5, 3.0, 3.5, 4.0, 4.5, 5.0, 6.0, 8.0, 10.0, 12.0, 24.0, 36.0, 48.0 and 72 h (after drug administration) in coded centrifuge tubes containing EDTA. Blood samples were centrifuged immediately and the plasma was separated into duplicate polypropylene tubes and stored the samples at -20°C until analysis. All the physical and medical examinations of all the volunteers were performed throughout the study period starting from the screening of the volunteers. The following pharmacokinetic parameters were considered for evaluation of the bioequivalence study between test and reference products – major parameters (according to FDA), C_max_ – observed maximum plasma concentration; AUC_last_ – area under plasma concentration/time plot until the last quantifiable value; auxiliary parameters, T_max_ – sampling time of the maximum plasma concentration; t-half – terminal elimination half life time; AUC_total_ – area under plasma concentration/time plot extrapolated to infinity. All the pharmacokinetic parameters were determined by WinNonlin software (version 6.3) from Pharsight, USA. The analysis of variance was performed on the pharmacokinetic parameters. Finally, the 90% CIs of the pharmacokinetic parameters characterizing the tested/reference products were determined.

## Results & discussion

### Method development

To develop and validate a selective and rapid assay method for simultaneous quantification of both EPL and EPLT in human plasma, it is important to optimize chromatographic conditions, mass spectrometric parameters and the extraction technique.

### Mass spectrometry

MS parameters having electrospray as the ionization source were optimized by injecting standard analyte solution of 100 ng/ml into the LC–MS/MS and operating in the multiple reaction monitoring mode. MS parameters were tuned in both positive and negative ionization modes for EPL, EPLT and TBM (IS). Positive ionization mode has shown much higher signal intensities than those in negative ionization mode, since the analytes and IS have the ability to accept protons. EPL, EPLT and TBM (IS) showed the formation of predominant protonated [M+H]^+^precursor ions at *m/z* 377.2, 349.1 and 271.1, respectively, in Q1 MS full scan spectra. The most copious ions found in the product ion mass spectrum were *m/z* 234.2, 206.1 and 155.0 for EPL, EPLT and TBM (IS), respectively (Figure 1). Observing maximum response of the product ion the source-dependent and compound-dependent parameters (Tables 1 & 2) were determined. ESI was selected compared with the atmospheric chemical ionization as the ionization source as it gave high spectral response for both the analytes and the regression curves obtained were linear. The ESI source provided reliable data on method development, validation and for quantitation of samples from human volunteers.

### Liquid chromatography

Since EPL and EPLT have almost similar structure and physicochemical properties, it was not difficult to optimize chromatographic conditions including mobile phase selection, flow rate, column type and injection volume that result sharp peak shape and adequate response. EPL and EPLT have less structural variations with p*K*_a_ (strongest acidic) of 3.0 and 2.97, polar surface area of 95.9 and 106.9, respectively [36]. Solvents such as acetonirtile, methanol in different ratio with buffers such as ammonium acetate, ammonium formate as well as acid additives such as formic acid and acetic acid as an enhancer in varying strength were tried. After several trials it was observed that gradient system using 0.1% formic acid in methanol at pump B and 0.1% formic acid in Milli Q water at pump A as the mobile phase was most appropriate to give best sensitivity, efficiency and peak shape. Formic acid helped to improve the peak shape and spectral response. Different kinds of columns or stationary phases such as C18 of PhenomenexKinetex, C18 and CN of Agilent were tried for checking better selectivity and sensitivity during method development. The use of a short chromatographic column (PhenomenexKinetex C18, 100A 50 × 3 mm, 5 µm particle size) and optimized flow rate (0.50 ml/min.) helped in the separation and elution of all the analytes of interest in a very short time with good peak shape and response, whereas the use of Agilent C18 and CN resulted in poor peak shape and in response also. So finally this Phenomenex Kinetex C18 column was selected for our analysis. The total chromatographic run time was 3.0 min for each run.

### Selection of internal standard

In the initial phase, several compounds, such as metformin, TBM, losartan etc. were investigated and finally TBM was concluded as a best choice of internal standard (IS). It has shown no interference with the performance of the developed method and the intensity of molecular ion peak in mass spectrometric analysis remained unaffected as compared with others (Figures 2 & 3). The flow rate 0.5 ml/min was optimized on basis of the retention time of analytes and gradient elution provides faster separation with the chromatographic conditions as described above.

## Method validation

### Selectivity & LLOQ

Representative chromatograms of the double blank and blank plasma samples spiked with IS are shown in Figure 2. Also, blank plasma spiked with 1 ng/ml of EPL and EPLT at their LLOQs together with 100 ng/ml of the TBM (IS) are shown in Figure 2. The retention time for EPL, EPLT and TBM (IS) were found 2.13, 2.09 and 2.23 min (Figure 3), respectively. Inspection of chromatograms reveled that no significant interference from the plasma matrix was observed at the retention times of analytes or IS indicate the selectivity or specificity of the method. In the present assay, LOD were 0.25 and 0.80 ng/ml in plasma and LLOQ was found 1 ng/ml in plasma for both EPL and EPLT respectively, with an injection volume of 10 µl.

### Accuracy & precision

Summary of accuracies and precisions for intra- and inter-day runs for all analytes were shown in Table 3. Mean accuracies of the intraday QC samples ranged from 103 to 113% and 93–103%, respectively, for EPL and EPLT while that of interday samples ranged from 100 to 106% and 96 to 99%, respectively, for EPL and EPLT. The CV for all intraday QC samples ranged from 3.4 to 11.8% and 3.1–10.3% for EPL and EPLT, respectively. The CV for interday ranged from 7.7 to 10.9% for EPL and 6.7 to 13.7% for EPLT. These data ensure that the present method has a satisfactory accuracy, precision and reproducibility for the quantification of both EPL and EPLT in human plasma. In general the method is accurate and precise as per the requirements of the FDA and EMA guidelines [26,32].

### Recovery & matrix effect

The recovery study of protein precipitation method was performed on three QCs concentrations levels as shown in Table 4 that ranges from 96.5 to 99.3% and 94.2 to 98.8%, respectively, for EPL and EPLT. The recovery of both analytes is sufficiently high and data indicated good sensitivity, precision and accuracy.

No significant matrix effect was found by comparing the AUC ratios of the extracted QCs and IS with the AUC of unextracted QCs and IS obtained from the injecting raw solution prepared at similar concentration levels elaborated in Table 5.

### Linearity of the assay

The assay result demonstrated a good linear response over the range 1–500 ng/ml for both EPL and EPLT in human plasma. The regression equations (n = 8) were y = 0.02697x +0.007590 (r = 0.99743) for EPL and y = 0.00524x +0.0014317 (r = 0.99727) for EPLT, where y is the peak area ratio of analyte to IS and x is the nominal plasma concentration. At calibration curve concentrations, the % CV and accuracy values ranged from 0.51 to 9.99% and 93.38 to 105.53% for EPL and from 2.67 to 28.39% and 96.49 to 109.80% for EPLT.

### Stability

At the various QC concentrations, values of percent accuracy and coefficient of variation for bench-top, autosampler, freeze–thaw and long-term stability evaluation were deviated within the accepted range as summered in Tables 6 & 7 for both EPL and EPLT, respectively. The autosampler stability was found stable for at least 24 h at 24°C temperature. Both analytes in control human plasma at room temperature were found stable at least for 8 h (bench top stability), stable in autosampler for 24 h and for minimum of three freeze and thaw cycles. Spiked plasma samples, stored at -20°C for long-term stability experiment, were found stable for minimum of 30 days. In general, the analytes were found stable under the different testing conditions with acceptable percent accuracy and CVs.

### Application of LC–MS/MS method in bioequivalence study

The method was applied to evaluate plasma samples from 30 healthy volunteers after an oral administration of EPL 20 mg tablet. The mean plasma concentration time profile of EPL and EPLT in human volunteers is depicted in Figure 4 and the mean estimated pharmacokinetic parameters derived from the plasma concentration profiles are summarized in Table 8. No statistically significant differences were found in any parameter between the two formulations. The mean log transformed ratios of the parameters and the 90% CI values were all within the required ranges confirming the bioequivalence of the test and reference products.

### Comparison to other methods

This bio-analytical method has several advantages over the previously reported methods [9,14,16–17,20,25,37]. Sample preparation is simpler and the chromatographic column and IS used are commercially available. The procedure for sample preparation and extraction are rapid and inexpensive due to use simple protein precipitation technique by acetonitrile. The method is very sensitive (LLOQ is very low) and selective and also, the extraction recoveries of both EPL and EPLT are better (more than 90%) compared with other published methods. The method prevented phospholipid-based matrix effect on the analytes of interest. So, the results of matrix effect showed that ion suppression or enhancement from the plasma matrix components was abolished. Another advantage of this method is the use of a gradient mobile phase of simple composition with reduction in run time of analysis (3.0 min), which allows determination of the analytes more precisely. The stability studies have been carried out extensively. Finally, the validated method was applied to pharmacokinetic and bioequivalence study of both EPL and EPLT in Indian volunteers.

## Conclusion

The main objective of this work was to develop a simple, specific, rapid, highly sensitive, rugged and high-throughput bio-analytical method for simultaneous determination of an active metabolite EPLT with its prodrug EPL in human plasma. The advantage of using a simple protein precipitation technique in the present work is due to high extraction recovery, minimization of the sample extraction time, present method has been used only 300 µl of human plasma and hence to reduce the amount of blood withdrawn from volunteers during study, proposed method is applicable in any strength of the drug for bioequivalence studies, it gives cleaner and consistent extraction without any significant matrix effect, because of rapid sample preparation technology and short chromatographic run time (3.0 min), large numbers of pharmacokinetic samples can be analyzed.

Therefore, the bio-analytical method discussed above is precise, accurate, reproducible, stable and robust enough to enable pharmacokinetic, bioavailability or bioequivalence studies on a long-term basis. Also, this bio-analytical method is simple, selective, rapid, specific, highly sensitive and fully validated by FDA and EMA guidelines and the resulting pharmacokinetic parameters would be helpful to provide some guidance to clinical application and investigation.

## Future perspective

In 5–10 years’ time, we believe that this method will be used to evaluate compartmental pharmacokinetic study under the framework of long-term therapeutic effect. This will also help to establish the therapeutic efficacy of the drug EPL. Pharmacokinetics or quantitative study of active metabolites in plasma is a very crucial part of drug development studies as sometimes it shows that the active metabolites are more pharmacologically active substances than the prodrug after entering the systemic circulation. In India, the *in vivo* pharmacokinetics study of active metabolites is limited. Thus, in the near future, this kind of approach will enrich the potentiality of pharmacokinetics or quantitative study of the active metabolites in plasma.

**Table 1.** **Optimized mass parameters for analytes and internal standard.**

**Analytes**	**Compound-dependent parameters**				
	**CE**	**DP**	**CEP**	**CXP**	**EP**
Enalapril	30	30	29.73	4	11
Enalaprilat	30	30	29.08	4	11
Tolbutamide (IS)	30	30	27.29	4	11

CE: Collision energy; CEP: Cell entrance potential; CXP: Collision cell exit potential; DP: Declustering potential; EP: Entrance potential; IS: Internal standard.

**Table 2.** **Optimized source-dependent parameters.**

**Source-dependent parameters**	**Value**
Nebulizer gas (gas 1)	55
Heater gas (gas 2)	45
Ion spray voltage	5000
Collision activated dissociation gas	6
Curtain gas	30
Source temperature	400
Focusing potential	400

**Table 3.** **Intra- and inter-day precision and accuracy data for enalapril and enalaprilat in human plasma (n = 6).**

	**Intraday (within run)**				**Interday (between run)**			
	**Mean concentration (ng/ml)**	**SD**	**CV (%)**	**Accuracy (%)**	**Mean concentration (ng/ml)**	**SD**	**CV (%)**	**Accuracy (%)**
**Enalapril**								
LQC (3 ng/ml)	3.0933	0.1075	3.4738	103.1111	3.0113	0.3226	10.7128	100.3750
MQC (200 ng/ml)	223.9300	26.4513	11.8123	111.9650	207.6733	22.7122	10.9365	103.8367
HQC (400 ng/ml)	453.7200	35.9618	7.9260	113.4300	425.1263	32.8410	7.7250	106.2816
**Enalaprilat**								
LQC (3 ng/ml)	3.1150	0.3038	9.7515	103.8333	2.8988	0.3990	13.7661	96.6250
MQC (200 ng/ml)	187.9250	19.3644	10.3043	93.9625	199.1229	23.3814	11.7422	99.5615
HQC (400 ng/ml)	393.6150	12.2396	3.1095	98.4038	399.9475	27.1177	6.7803	99.9869

HQC: High quality control; LQC: Low quality control; MQC: Medium quality control.

**Table 4.** **Absolute recovery of enalapril and enalaprilat from plasma (n = 6).**

	**Spiked concentration (ng/ml)**	**Mean concentration (ng/ml)**	**Recovery (%), mean ± SD**
Enalapril	3	2.8043	96.5478 ± 15.6653
	200	204.5113	97.8200 ± 5.7916
	400	400.2418	99.3747 ± 8.2698
Enalaprilat	3	2.94500	98.8382 ± 6.7260
	200	196.4750	94.2010 ± 10.2092
	400	402.69233	98.5074 ± 10.9687

**Table 5.** **Matrix effect of enalapril and enalaprilat calculated from six different source of human plasma.**

**Analytes**	**Matrix effect (ME %)^†^**						**Mean**	**SD**	**% CV**
	**SET 1^‡^**	**SET 2**	**SET 3**	**SET 4**	**SET 5**	**SET 6**			
**Enalapril**									
LQC (3 ng/ml)	95.8510	104.6398	106.0474	92.9591	109.0460	94.7069	100.5417	6.8260	6.7893
MQC (200 ng/ml)	113.3058	105.8869	90.9534	112.3145	91.5637	96.4744	101.7498	10.1036	9.9299
HQC (400 ng/ml)	103.3769	92.1597	99.1176	105.8241	94.9351	97.4031	98.8028	5.1258	5.1879
**Enalaprilat**									
LQC (3 ng/ml)	106.9987	96.3672	110.2372	94.5207	95.3574	105.6815	101.5271	6.8825	6.7789
MQC (200 ng/ml)	94.9127	109.5726	101.5992	98.4407	105.2650	97.6180	101.2347	5.4176	5.3515
HQC (400 ng/ml)	95.3538	113.0894	91.0844	106.6942	90.7440	95.3352	98.7168	9.1079	9.2263

^†^ME% expressed as the ratio of the mean peak area of an analyte spiked post extraction to the mean peak area of the same analyte standards multiplied by 100. A value of >100% indicates ionization enhancement, and a value of <100% indicates ionization suppression.

^‡^SET 1–6 represent the ME% from six different source of human plasma.

HQC: High quality control; LQC: Low quality control; ME: Matrix effect; MQC: Medium quality control.

**Table 6.** **Stability of enalapril under different storage conditions (n = 6).**

**Serial number**	**Storage conditions**	**QC samples (ng/ml)**	**Mean (ng/ml)**	**SD**	**CV (%)**	**Accuracy (%)**
1	Autosampler stability 24 h at 24°C	3	2.7500	0.4934	17.9402	92.6446
		200	200.9350	12.6188	6.2800	100.4675
		400	384.6733	15.7191	4.0863	92.6098
2	Freeze and thaw stability at -20°C (3 cycles)	3	2.7200	0.4528	16.6459	91.6339
		200	189.7283	16.5433	8.7195	94.7244
		400	388.3750	31.0460	7.9938	93.5010
3	Bench top stability for 8 h	3	2.7350	0.3092	11.3068	92.1392
		200	200.9567	16.9404	8.4299	100.3303
		400	412.2483	23.03918	5.5887	99.2485
4	Long-term stability at -20.0°C for 30 days	3	2.7000	0.5956	22.0599	90.9601
		200	197.6167	24.1216	12.2062	98.6628
		400	393.2767	13.0709	3.3236	94.6810

**Table 7.** **Stability of enalaprilat under different storage conditions (n = 6).**

**Serial number**	**Storage conditions**	**QC samples (ng/ml)**	**Mean (ng/ml)**	**SD**	**CV (%)**	**Accuracy (%)**
1	Autosampler stability 24 h at 24°C	3	2.6650	0.2892	10.8514	98.5516
		200	187.7900	31.1432	16.5840	93.8950
		400	365.4017	18.0123	4.9295	92.5255
2	Freeze and thaw stability at -20°C (3 cycles)	3	2.3750	0.5358	22.5595	87.8274
		200	177.4900	18.3801	10.3556	95.3222
		400	348.4317	61.8666	17.7557	88.2284
3	Bench top stability for 8 h	3	2.8833	0.3502	12.1460	106.6256
		200	190.4450	22.0556	11.5811	102.2798
		400	387.3033	20.70389	5.3457	98.0713
4	Long-term stability at -20.0°C for 30 days	3	2.4967	0.4318	17.2939	92.3267
		200	196.7650	8.2097	4.1723	105.6740
		400	394.0400	12.4395	3.1569	99.7772

**Table 8.** **Pharmacokinetic parameters of healthy human volunteers with the test and reference preparation (n = 30).**

**Pharmacokinetic parameters**		**Enalapril 20 mg**		**Enalaprilat**	
		**Reference preparation**	**Test preparation**	**Reference preparation**	**Test preparation**
C_max_ (ng/ml)	Mean	254.3130 ± 16.1820	249.7880 ± 15.4990	121.3210 ± 10.7660	117.9480 ± 11.5540
t_max_ (h)	Mean	0.6810 ± 0.2700	0.8060 ± 0.2530	2.2500 ± 0.4870	2.7000 ± 0.5350
AUC _0–72_ (ng. h/ml)	Mean	317.4790 ± 16.4780	315.2940 ± 22.3340	1043.1070 ± 50.2960	1052.9040 ± 60.5320
AUC _0-∞_ (ng. h/ml)	Mean	319.8340 ± 16.4150	317.7470 ± 22.2500	1086.8190 ± 53.3050	1099.8880 ± 56.1890
k_el_ (h^-1^)	Mean	0.4970 ± 0.0210	0.4860 ± 0.0200	0.0750 ± 0.0040	0.0730 ± 0.0050
t_1/2_ (h)	Mean	1.3960 ± 0.0590	1.4290 ± 0.0580	9.2490 ± 0.5320	9.5510 ± 0.6190
Relative bioavailability (%)		100	99.31	100	100.94

C_max_: Maximum plasma concentration.

t_max_: time require to achieve maximum concentration.

AUC_0–72_: area under the plasma concentration time curve from time zero to 72 h.

AUC_0–∞_: area under the plasma concentration-time curve from time zero to infinity.

K_el_: elimination rate constant.

t_1/2_: elimination half life.

**Executive summary**

A simple, selective, rapid, specific, highly sensitive and high-throughput tandem mass spectroscopic method has been developed and validated for simultaneous determination of enalapril (EPL) and its active metabolite enalaprilat (EPLT) in human plasma.
The LLOQ obtained indicates the high sensitivity of the described method of analysis, which is useful for pharmacokinetic or bioequivalence studies.
Sample extraction procedure was performed by a simple protein precipitation technique.
The results of matrix effect showed that ion suppression or enhancement from the plasma matrix components was abolished.
The stability studies have been carried out extensively and the results were deemed acceptable.
The method was applied to compare the bioavailability of two formulations of EPL by conducting the single oral dose, open label, randomized, two period, two sequence, crossover study of 30 healthy Indian male volunteers.
